# DiSCO: deconvoluting spatial transcriptomics via combinatorial optimization with a foundational diffusion model

**DOI:** 10.1093/bib/bbag207

**Published:** 2026-05-08

**Authors:** Jing Liu, Yahao Wu, Limin Li

**Affiliations:** School of Mathematics and Statistics, Xi’an Jiaotong University, Xianning West 28, 710049 Shaanxi, China; School of Mathematics and Statistics, Xi’an Jiaotong University, Xianning West 28, 710049 Shaanxi, China; School of Mathematics and Statistics, Xi’an Jiaotong University, Xianning West 28, 710049 Shaanxi, China

**Keywords:** diffusion model, foundational model, combinatorial optimization, deconvolution

## Abstract

Deciphering the cellular composition of spatial spots in spatial transcriptomics (ST) data is fundamental for elucidating the heterogeneity of tissue spatial structures. However, existing models often require retraining for each new deconvolution task, reflecting limitations in both generalization performance and computational efficiency. To address this problem, we design a foundational **di**ffusion model to deconvoluting **s**patial transcriptomics based on **c**ombinatorial **o**ptimization, termed DiSCO. DiSCO formulates the deconvolution of ST data as a task-specific deconvolutional combinatorial optimization (CO) problem, wherein single cells (SCs) are assigned to spatial spots to optimally preserve the gene expression profiles of each spot. DiSCO introduces a bipartite graph diffusion model as an optimization solver, specifically designed to be generalizable to any new deconvolutional CO problem. Pretrained on a large number of deconvolution tasks using gene expression profiles of both SCs and spatial spots as inputs, DiSCO learns the distribution of true solutions and generates approximate solutions through sampling, thereby enabling the determination of the cellular composition for each spot. As a generalizable deconvolution solver, the DiSCO is evaluated by experiments on both simulated datasets and real datasets, demonstrating that the pretrained DiSCO model performs effectively and efficiently on datasets with varying resolutions and different numbers of genes, thus highlighting its capacity to effectively generalize to diverse datasets.

## Introduction

Spatial transcriptomics (ST) [1] is a newly emerging technique that enables the mapping of gene expression profiles while preserving spatial structural information. This technology provides novel tools for understanding the structure and function of cells and tissues, and has been applied to biomedical studies of the brain, lymph nodes, and heart in both mice and humans. However, current ST sequencing technologies either do not achieve single-cell (SC) resolution ($10\times $ Genomics Visium platform, Slide-seqV2 [2]) or have very low gene throughput (seqFISH+ [3], MERFISH [4]). In contrast, SC RNA sequencing technologies can analyze a large number of genes at SC resolution but do not provide spatial information. Both sequencing technologies have distinct advantages. Therefore, integrating both types of sequencing data to deconvolute ST can facilitate a more comprehensive understanding of cellular mechanisms in an organism.

Existing cell type deconvolution methods typically use SC data as a reference to decompose gene expression at a spot into linear combinations of different cell types. One such method, Spotlight [5], uses non-negative matrix factorization (NMF) and non-negative least-squares regression (NNLS) to infer the cell type composition of each spot. Cell2location [6] employs a generalized Bayesian model to map cell types, while Tangram [7], a mapping-based approach, proposes a deep-learning-based method to learn the mapping of SCs to a spot. Redeconve [8] and scDOT [9], also mapping-based approaches, use a regularized non-negative regression model and an optimal transport model, respectively, to learn the mapping relationship between paired data. Methods such as DSTG [10] and SD$^{2}$ [11] first construct pseudo-points, learn their cellular composition via a graph neural network, and generalize to actual deconvolution tasks. All these methods require the model to be trained or optimized from scratch for each new deconvolution task. This means that when new ST data need to be deconvolved, the trained model, or optimized parameters from the previous dataset cannot be directly applied, limiting their generalization performance.

In this work, we aim to propose a foundational deconvolution model that can be generalized to new deconvolutional problems, from the aspects of combinatorial optimization (CO) [12]. We mathematically formulate the deconvolution problem as a CO problem, which assigns SCs to different spatial spots. It has been challenging to solve CO problems. Although literature exists on the application of deep learning techniques to CO, such as difusco [13] addressing the shortest path problem via diffusion models [14], and NHDE [15] solving multi-objective optimization problems using a heterogeneous graph attention model, the optimization objectives in these methods primarily focus on edges within simple graphs. Therefore, these methods cannot be directly applied to edges between nodes in bipartite graphs, which are inherently heterogeneous, and this is particularly critical for tasks such as the cell type deconvolution problem. More importantly, the pure CO solver could not directly used for deconvoluting ST.

With a large number of SC resolution spatial transcriptomic slices, DiSCO learns an optimization solver by a bipartite graph diffusion model, which is trained to maximize the likelihood of the optimal solutions with different regularization terms for spatial deconvolution problems. By carefully designing the diffusion and denoising process, along with a bipartite graph neural network (BGNN) as the backbone in DiSCO, and the loss function, DiSCO is pretrained to work as a generalizable solver for deconvolution tasks. It is pretrained on a large set of deconvolution tasks, using gene expression profiles from both SCs and spatial spots as inputs. Through this process, DiSCO learns the underlying distribution of optimal solutions, enabling it to generate approximate solutions via sampling, which in turn facilitates the accurate determination of cellular composition for each spatial spot. Experiments over various datasets show that the pretrained DiSCO can be directly applied to new deconvolution tasks with good generalization performance. The DiSCO is freely available in https://github.com/LiminLi-xjtu/DiSCO.

## Materials and methods

### Overview of DiSCO

Given a data pair $\mathcal{D}=(\boldsymbol{X},\boldsymbol{Y})$, where $\boldsymbol{X}$ represent the referenced gene expression matrix for SCs with dimension $ {G \times N_{c}} $, and $\boldsymbol{Y}$ denotes the gene expression matrix for spatial spots with dimension $ {G \times N_{s}} $, the goal of SC level deconvolution is to find an assignment matrix $\boldsymbol{A}$ with dimensions $N_{c} \times N_{s}$, where each element $\boldsymbol{A}_{i,j}=1$ indicates that cell $i$ has been assigned to spatial spot $j$.

DiSCO addresses this problem by learning a CO solver, to determine an optimal assignment decision $\boldsymbol{A} \in \{0,1\}^{N_{c} \times N_{s}}$, where each row containing exactly one 1, such that $\boldsymbol{Y}$ is best approximated by $\boldsymbol{X}\boldsymbol{A}$. Based on the optimal assignment, the cellular composition of each spatial spot, including the number of cells and their corresponding cell types, can be accurately determined.

**Figure 1:**
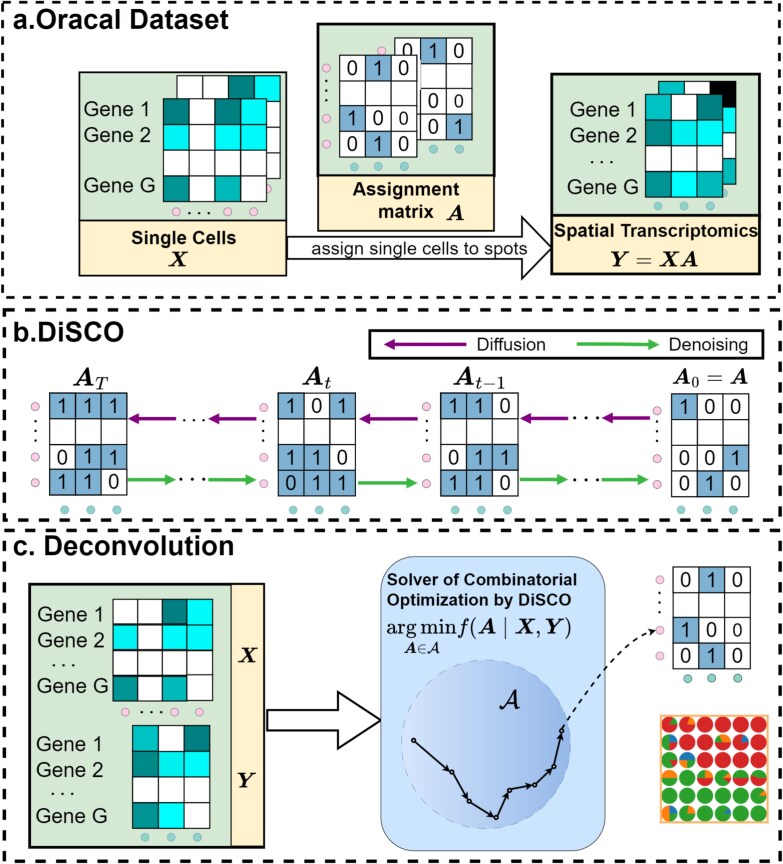
The overview of DiSCO. (a) The oracal dataset for the specific deconvolution task: $\boldsymbol{X},\boldsymbol{Y} $ denote the gene profile from SCs and the spots, respectively. (b) The process of diffusion and denoising in DiSCO. (c) DiSCO searches for an approximate solution $\boldsymbol{A}$ in the candidate solution space $ \mathcal{A}$ conditional on $\boldsymbol{X}, \boldsymbol{Y}$.

DiSCO reformulates the solver as a sequential recovery process characterized by a Markovian structure over bipartite graph diffusion. The model is trained on the constructed *oracle data*  $\{\mathcal{T}^{s}\}_{s=1}^{S}$, where each $\mathcal{T}^{s} = (\boldsymbol{X}^{s}, \boldsymbol{Y}^{s}, \boldsymbol{A}^{s})$ satisfies $\boldsymbol{Y}^{s} \approx \boldsymbol{X}^{s} \boldsymbol{A}^{s}$ for a known ground-truth assignment $\boldsymbol{A}^{s}$, as illustrated in Fig. 1a. Through a diffusion–denoising mechanism, the model progressively refines random initial solutions into feasible assignments, thereby asymptotically approximating the distribution of optimal assignments. This process also captures the biological transition of cell distributions from a disordered to an ordered state. Figure 1b illustrates the forward diffusion and reverse denoising processes. The forward process gradually corrupts the ground-truth assignment into noise, following a common distribution across all instances, whereas the reverse process is trained to reconstruct the solution distribution $p(\boldsymbol{A}\mid \boldsymbol{X},\boldsymbol{Y})$ from the consistent noise distribution. This reverse process is implemented via a BGNN—an anisotropic GNN that we design to capture the structural dependencies within bipartite graphs. Finally, Fig. 1c depicts the inference procedure for deconvolution on a new instance. Given observed data $(\boldsymbol{X},\boldsymbol{Y})$, DiSCO first samples an initial assignment from a prior distribution, then applies the reverse denoising process, guided by $(\boldsymbol{X},\boldsymbol{Y})$, to recover an approximate solution $\boldsymbol{A}$, and accomplish the deconvolution task.

### Formulating deconvolution as a combinatorial optimization

For a given pair of ST and SC data $\mathcal{D}=(\boldsymbol{X},\boldsymbol{Y})$, we define the space of candidate assignment matrices as 


\begin{align*} & \mathcal{A}_{D} = \left\{ \boldsymbol{A} \in \{0,1\}^{N_{c} \times N_{s}} \;\middle|\; \text{each row of } \boldsymbol{A} \text{ contains exactly one } 1 \right\}, \end{align*}

where $N_{c}$ and $N_{s}$ denote the number of SCs and spatial spots, respectively. The deconvolution problem can then be formulated as the CO: 


(1) \begin{align*}& \begin{aligned} \boldsymbol{A}^{*} &= \arg\min_{\boldsymbol{A} \in \mathcal{A}_{\mathcal{D}}} \operatorname{cost}_{\mathcal{D}}(\boldsymbol{A}) + \operatorname{valid}_{\mathcal{D}}(\boldsymbol{A})\\ &=\arg\min_{\boldsymbol{A} \in \mathcal{A}_{\mathcal{D}}} f(\boldsymbol{A}\mid\mathcal{D}), \end{aligned}\end{align*}

where 


\begin{align*} &\operatorname{cost}_{\mathcal{D}}(\boldsymbol{A}) = \|\boldsymbol{X}\boldsymbol{A} - \boldsymbol{Y}\|_{F}\end{align*}

measures the task-specific discrepancy between the aggregated SC profiles and the observed spatial transcriptomic data, and $\operatorname{valid}_{D}(\boldsymbol{A})$ is a feasibility term that enforces the row-wise one-hot constraint: it returns 0 if $\boldsymbol{A} \in \mathcal{A}_{D}$ and $\infty $ otherwise. Equivalently, the assignment matrix $\boldsymbol{A}$ can be represented as a bipartite graph, where nodes correspond to SCs and spatial spots, and edges indicate the assignment of cells to spots.

Importantly, this CO problem is inherently task-specific: each deconvolution task defined by a distinct $\mathcal{D}=(\boldsymbol{X},\boldsymbol{Y})$ yields a different objective function and solution space for the optimal assignment $\boldsymbol{A}^{*}$. Consequently, constructing a solver that generalizes across all possible deconvolution tasks is highly challenging.

### Oracle data construction

DiSCO constructs training datasets *oracle data*  $\{\mathcal{T}^{s}\}_{s=1}^{S}$ for spatial deconvolution, where each $\mathcal{T}^{s} = (\boldsymbol{X}^{s}, \boldsymbol{Y}^{s}, \boldsymbol{A}^{s})$. In practice, scCube [16] is first used to generate spatially resolved SC expression profiles $\boldsymbol{X}^{s}$. The data are subsequently down-sampled to a lower spatial resolution by delineating spots, and spot-level expressions $\boldsymbol{Y}^{s}$ are constructed by aggregating nearby cells based on a given assignment matrix $\boldsymbol{A}^{s}$. This procedure yields 10 000 supervised input–solution instances $\{\mathcal{T}_{s}\}_{s=1}^{S}= \{(\boldsymbol{X}^{s},\boldsymbol{Y}^{s},\boldsymbol{A}^{s})\}_{s=1}^{S}$. For each instance, we simulated 1224 SC expression profiles across four distinct cell types (endothelial cells, erythrocytes, fibroblasts, and professional antigen-presenting cells) with 2000 genes, which were subsequently aggregated into $\sim $255 spots. In the original work [16], scCube showed significant diversity in generating simulated datasets, encompassing various spatial patterns, resolutions, spot arrangements, and tissue slice dimensions. Its effectiveness in benchmarking spot deconvolution has been validated through comparative experiments. Furthermore, DiSCO is designed to learn a solver for CO without explicitly using cell-type or spatial structural information, the Oracle data in these simulations provides sufficient information for the model to acquire this capability. The training set, validation set, and test set were divided in a 9000/900/100 scale to ensure that instances in the test set were unseen during training.

### DiSCO model with bipartite graph diffusion

We propose a DiSCO model that formulates the optimization in Equation (1) on a bipartite graph within the framework of Discrete Denoising Diffusion Probabilistic Models (D3PMs) [17]. This bipartite design enables efficient diffusion over the structured *oracle data*  $\mathcal{T}^{s} = (\boldsymbol{X}^{s}, \boldsymbol{Y}^{s}, \boldsymbol{A}^{s})$, thereby enhancing both interpretability and convergence. In this formulation, DiSCO aims to recover the ground-truth optimal solution $\boldsymbol{A}^{s}$ for a given pair $(\boldsymbol{X}^{s}, \boldsymbol{Y}^{s})$. Following the diffusion modeling convention, we denote the ground-truth assignment as $\boldsymbol{A}_{0} = \boldsymbol{A}^{s}$, representing the target solution of the deconvolutional CO problem.

#### Discrete diffusion step

To diffuse the bipartite graph $\boldsymbol{A}_{0}$ into a random one from a discrete uniform distribution, the diffusion step is defined by a series of discrete Markov processes, which progressively add Bernoulli noise to the bipartite graph. Specifically, at time step $t$, we define the transition probability as: $q\left (\boldsymbol{A}_{t} \mid \boldsymbol{A}_{t-1}\right )=\operatorname{Cat}\left (\boldsymbol{A}_{t} ; \boldsymbol{p}=\tilde{\boldsymbol{A}}_{t-1} \boldsymbol{Q}_{t}\right )$, where $\operatorname{Cat}$ denotes a categorical distribution, $\tilde{\boldsymbol{A}}_{t-1} \in \left \{0, 1\right \}^{(N_{c} N_{s}) \times 2}$ is transformed from $\boldsymbol{A}_{t-1} \in \left \{0, 1\right \}^{N_{c} \times N_{s}}$, and $\boldsymbol{Q}_{t}=\left [\begin{array}{@{}cc@{}}\left (1-\beta _{t}\right ) & \beta _{t} \\ \beta _{t} & \left (1-\beta _{t}\right )\end{array}\right ] $is the transition probability matrix. The parameter $\beta _{t}$ controls the degree of noise added at step $t$, As $\beta _{t}$ approaches zero, the probability of each state transfer decreases. $\left \{\beta _{t}\right \}$ is generally set up as an increasing series from 0, thereby guaranteeing the validity of $\prod _{t=1}^{T}\left (1-\beta _{t}\right ) \approx 0$, and $q\left (\boldsymbol{A}_{t} \mid \boldsymbol{A}_{0}\right )=\operatorname{Cat}\left (\boldsymbol{A}_{t} ; \boldsymbol{p}=\tilde{\boldsymbol{A}}_{0} \overline{\boldsymbol{Q}}_{t}\right )$, where $\overline{\boldsymbol{Q}}_{t}=\boldsymbol{Q}_{1} \boldsymbol{Q}_{2} \ldots \boldsymbol{Q}_{t}$, such that $\boldsymbol{A}_{T}$ follows a discrete uniform distribution.

#### Discrete denoising step

The denoising process evolves a noisy bipartite graph $\boldsymbol{A}_{T}$ to an approximate solution $\tilde{\boldsymbol{A}}_{0}$.

In the denoising process, we first initialize $\boldsymbol{A}_{T}$ by sampling from the uniform distribution, and then the posterior at time $t-1$ can be obtained by Bayes’ theorem: 


(2) \begin{align*}& \begin{aligned} q\left(\boldsymbol{A}_{t-1} \mid \boldsymbol{A}_{t}, \boldsymbol{A}_{0}\right)&=\frac{q\left(\boldsymbol{A}_{t} \mid \boldsymbol{A}_{t-1} \boldsymbol{A}_{0}\right) q\left(\boldsymbol{A}_{t-1} \mid \boldsymbol{A}_{0}\right)}{q\left(\boldsymbol{A}_{t} \mid \boldsymbol{A}_{0}\right)}\\ &=\operatorname{Cat}\left(\boldsymbol{A}_{t-1} ; \boldsymbol{p}=\frac{\tilde{\boldsymbol{A}}_{t} \boldsymbol{Q}_{t}^{\top} \bigodot \tilde{\boldsymbol{A}}_{0} \overline{\boldsymbol{Q}}_{t-1}} {\tilde{\boldsymbol{A}}_{0} \overline{\boldsymbol{Q}}_{t} \tilde{\boldsymbol{A}}_{t}^{\top}}\right), \end{aligned}\end{align*}

where $\bigodot $ denotes the element-wise multiplication, and $\tilde{\boldsymbol{A}}_{0}$ can be predicted by a graph neural network based on $\boldsymbol{A}_{0}$-parameterization: $\tilde{\boldsymbol{A}}_{0} \mid \boldsymbol{A}_{t} = \tilde{p}_{\theta }\left (\tilde{\boldsymbol{A}}_{0} \mid \boldsymbol{A}_{t}, \boldsymbol{X}^{s},\boldsymbol{Y}^{s} \right )$, so that the denoising process in Equation (2) can be formulated as: 


(3) \begin{align*}& \begin{aligned} q\left(\boldsymbol{A}_{t-1} \mid \boldsymbol{A}_{t}\right) &= p_{\theta}\left(\boldsymbol{A}_{t}-1 \mid \boldsymbol{A}_{t}, \boldsymbol{X}^{s},\boldsymbol{Y}^{s} \right) \\&= \sum_{\tilde{\boldsymbol{A}}_{0}} q\left(\boldsymbol{A}_{t-1} \mid \boldsymbol{A}_{t}, \tilde{\boldsymbol{A}}_{0}\right) \tilde{p}_{\theta} \left(\tilde{\boldsymbol{A}}_{0} \mid \boldsymbol{A}_{t}, \boldsymbol{X}^{s},\boldsymbol{Y}^{s}\right), \end{aligned}\end{align*}

where $\boldsymbol{X}^{s}, \boldsymbol{Y}^{s} $ corresponds to the task-specific information including the gene expression information of SC&ST, and $\tilde{p}_{\theta }$ is the GNN model we need to train. We also employ DDIM [18] to accelerate the denoising process inference: 


(4) \begin{align*}& q\left(\boldsymbol{A}_{\tau_{i}} \mid \boldsymbol{A}_{0}\right)=\operatorname{Cat}\left(\boldsymbol{A}_{\tau_{i}} ; \boldsymbol{p}=\boldsymbol{A}_{0} \overline{\boldsymbol{Q}}_{\tau_{i}}\right),\end{align*}

while the posterior can be computed by: 


(5) \begin{align*}& \begin{aligned} q\left(\boldsymbol{A}_{\tau_{i-1}} \mid \boldsymbol{A}_{\tau_{i}}, \boldsymbol{A}_{0}\right)&=\frac{q\left(\boldsymbol{A}_{\tau_{i}} \mid \boldsymbol{A}_{\tau_{i-1}}, \boldsymbol{A}_{0}\right) q\left(\boldsymbol{A}_{\tau_{i-1}} \mid \boldsymbol{A}_{0}\right)}{q\left(\boldsymbol{A}_{\tau_{i}} \mid \boldsymbol{A}_{0}\right)} \\ & =\operatorname{Cat}\left(\boldsymbol{A}_{\tau_{i-1}} ; \boldsymbol{p}=\frac{\tilde{\boldsymbol{A}}_{\tau_{i}} \overline{\boldsymbol{Q}}_{\tau_{i-1}, \tau_{i}}^{\top} \odot \tilde{\boldsymbol{A}}_{0} \overline{\boldsymbol{Q}}_{\tau_{i-1}}}{\tilde{\boldsymbol{A}}_{0} \overline{\boldsymbol{Q}}_{\tau_{i}} \ \tilde{\boldsymbol{A}}_{\tau_{i}}^{\top}}\right), \end{aligned}\end{align*}

where $\tau _{i-1} < \tau _{i}-1$, $\overline{\boldsymbol{Q}}_{t^{\prime }, t}=\boldsymbol{Q}_{t^{\prime }+1} \ldots \boldsymbol{Q}_{t}$.

### Bipartite graph denoising network

DiSCO takes the noisy bipartite graph $\boldsymbol{A}_{t}$ at time $t$ and task-specific instance $(\boldsymbol{X}^{s}, \boldsymbol{Y}^{s})$ as input and outputs the denoised bipartite graph $\boldsymbol{A}_{t-1}$. To better search for connecting edges between the nodes of a bipartite graph, we extend the Anisotropic Graph Neural Networks(AGNN) [19], a GNN that effectively encodes features for both nodes and edges, to bipartite graphs, resulting in the BGNN.

BGNN embeds the paired data $(\boldsymbol{X}^{s},\boldsymbol{Y}^{s})$ along with the noisy bipartite graph $\boldsymbol{A}_{t}$, and outputs $\boldsymbol{e}^{[0]}$, $\boldsymbol{u}^{[0]}$, and $\boldsymbol{v^{[0]}}$, respectively. Here $\boldsymbol{e}^{[0]}$ represents the latent embedding of bipartite graph at layer 0 and timestep $t$, while $\boldsymbol{u}$ and $\boldsymbol{v}$ denote the latent embeddings of cells and spots encoded by unshared parameters, respectively, to implicitly reduce the batch effects. Specifically, in layer $l$ of BGNN, it encodes temporal information into the noisy bipartite graph embeddings $\boldsymbol{e}^{\left [l\right ]}$ by temporal encoding: 


(6) \begin{align*}& \tilde{\boldsymbol{e}}^{\left[l+1\right]}=\boldsymbol{e}^{\left[l\right]}+\operatorname{ReLU}\left(\boldsymbol{W}\boldsymbol{h}_{t}^{\left[l\right]} \right),\end{align*}

where $\boldsymbol{h}_{t}^{0}$ is initialized as $\operatorname{MLP}_{t}(\boldsymbol{h}_{t})$, and $\boldsymbol{h}_{t}$ denotes the sinusoidal features [20] of denoising timestep $t$. We design a novel anisotropic message passing scheme for bipartite graph embeddings, which models cell and spot nodes with separate parameters to preserve heterogeneity. First, the edge embeddings are updated as follows: 


(7) \begin{align*}& \boldsymbol{e}_{ij}^{\left[l+1\right]} =\operatorname{ReLU}\left(\operatorname{LN}\left(\tilde{\boldsymbol{e}}_{ij}^{\left[l+1\right]}\boldsymbol{E}^{\left[l+1\right]} + \boldsymbol{u}_{i}^{\left[l\right]}\boldsymbol{M}^{\left[l\right]}+\boldsymbol{v}_{j}^{\left[l\right]}\boldsymbol{N}^{\left[l\right]} \right) \right),\end{align*}

where $\boldsymbol{E}^{\left [l\right ]}$, $\boldsymbol{M}^{\left [l\right ]}$, $\boldsymbol{N}^{\left [l\right ]} \in \mathbb{R}^{d\times d}$ are the learnable parameters of layer $l$ with dimension $d$, and $\operatorname{LN}$ represents Layer Normalization. Subsequently, the node embeddings are updated accordingly: 


(8) \begin{align*}& \begin{gathered} \boldsymbol{u}_{i}^{\left[l+1\right]}=\operatorname{ReLU}\left(\operatorname{LN}\left(\boldsymbol{u}_{i}^{\left[l\right]} \boldsymbol{O}^{\left[l\right]}+\mathcal{G}_{j}\left(\sigma\left(\boldsymbol{e}_{ij}^{\left[l+1\right]}\right) \odot \boldsymbol{v}_{j}^{\left[l\right]} \boldsymbol{P}^{\left[l\right]}\right)\right)\right),\\ \boldsymbol{v}_{j}^{\left[l+1\right]}=\operatorname{ReLU}\left(\operatorname{LN}\left(\boldsymbol{v}_{j}^{\left[l\right]} \boldsymbol{Q}^{\left[l\right]}+\mathcal{G}_{i}\left(\sigma\left(\boldsymbol{e}_{ij}^{\left[l+1\right]}\right) \odot \boldsymbol{u}_{i}^{\left[l\right]} \boldsymbol{R}^{\left[l\right]}\right)\right)\right), \end{gathered}\end{align*}

where $\boldsymbol{O}^{\left [l\right ]}$, $\boldsymbol{P}^{\left [l\right ]}$, $\boldsymbol{Q}^{\left [l\right ]}$, $\boldsymbol{R}^{\left [l\right ]} \in \mathbb{R}^{d\times d}$ are the learnable parameter of layer $l$, $\mathcal{G}$ denotes the aggregation function. The aggregated node embeddings and edge embeddings are then fed into a multi-layer perceptron (MLP) for modeling non-linear relationships. To reduce the vanishing gradient problem, residual connections are utilized. Finally, a 2-layer MLP serves as the bipartite graph decoder, which decodes the edge embeddings into a denoised graph.

### Loss function

The loss function consists of two components: the first part is the main objective function in the diffusion process, denoted as $L_{\mathrm{dpmm}}$, and the second part includes regularization terms specifically designed for the deconvolution task.

#### Diffusion loss function

Diffusion models optimizes $p_{\theta }\left (\boldsymbol{A}_{0}\right )$ to fit the distribution $q\left (\boldsymbol{A}_{0}\right )$ based on the variational upper bound on the negative log-likelihood: 


(9) \begin{align*} \begin{aligned} L_{\mathrm{vb}} = \ & \mathbb{E}_{q\left(\boldsymbol{A}_{0}\right)}[ \underbrace{D_{\mathrm{KL}}\left[q\left(\boldsymbol{A}_{T} \mid \boldsymbol{A}_{0}\right)| | p\left(\boldsymbol{A}_{T}\right)\right]}_{L_{T}} \\ &+\sum_{t=2}^{T} \underbrace{\mathbb{E}_{q\left(\boldsymbol{A}_{t} \mid \boldsymbol{A}_{0}\right)}\left[D_{\mathrm{KL}}\left[q\left(\boldsymbol{A}_{t-1} \mid \boldsymbol{A}_{t}, \boldsymbol{A}_{0}\right)| | p_\theta\left(\boldsymbol{A}_{t-1} \mid \boldsymbol{A}_{t}\right)\right]\right]}_{L_{t-1}} \\ & \underbrace{-\mathbb{E}_{q\left(\boldsymbol{A}_{1} \mid \boldsymbol{A}_{0}\right)}\left[\log p_\theta\left(\boldsymbol{A}_{0} \mid \boldsymbol{A}_{1}\right)\right]}_{L_{0}}]. \end{aligned}\end{align*}

It is noteworthy that if the value of $T$ is sufficiently large and a uniform distribution is selected as the prior $p(\boldsymbol{A}_{T})$, then the $L_{T}$ term is guaranteed to converge to $0$, regardless of the data distribution $q(\boldsymbol{A}_{0})$. Concurrently, it is acknowledged that the loss of cross-entropy > KL divergences, and consequently the inequality $L_{t-1} \leq \sum _{t=2}^{T} \mathbb{E}_{q\left (\boldsymbol{A}_{t} \mid \boldsymbol{A}_{0}\right )} \left [\log p_\theta \left (\boldsymbol{A}_{t}-1 \mid \boldsymbol{A}_{t}\right )\right ]$ is valid. In the reverse diffusion process, the $\boldsymbol{A}_{0}$-parameterization is employed in replacement of the $\boldsymbol{A}_{t}$-parameterization. Consequently, we derive the following inequality: 


(10) \begin{align*}& \begin{aligned} L_{\mathrm{vb}} &\leq -\sum_{t=1}^{T} \mathbb{E}_{q\left(\boldsymbol{A}_{t} \mid \boldsymbol{A}_{0}\right)}\left[\log p_\theta\left(\boldsymbol{A}_{t-1} \mid \boldsymbol{A}_{t}\right)\right] \\ &\propto -\sum_{t=1}^{T} \mathbb{E}_{q\left(\boldsymbol{A}_{t} \mid \boldsymbol{A}_{0}\right)}\left[\log \tilde{p}_{\theta}\left(\boldsymbol{A}_{0} \mid \boldsymbol{A}_{t}\right)\right] \\ &=L_{\mathrm{dpmm}}. \end{aligned}\end{align*}

We use the $L_{\mathrm{dpmm}}$ as the loss function to be optimized.

#### Regularization term

DiSCO formulates the cell-type deconvolution problems as CO problems, which are generally difficult to solve exact solution. While DiSCO is intended to find a satisfactory solution for a large number of solved deconvolutional problems. However, the main objective function is computed by the exact solution, which can only guide the model to learn the only exact solution. To enhance the robustness of DiSCO, the $L_{p}$ regular term is incorporated into the objective loss function. $L_{p}$ denotes the root mean square error ($\operatorname{RMSE}$) of cell-type proportion between the predicted and ground truth: 


(11) \begin{align*}& \begin{aligned} L_{p} = \sqrt{\frac{1}{N_{s}\cdot N_{ct}} \sum_{i=1}^{N_{s}} \sum_{j=1}^{N_{ct}} \left(p_{ij}-q_{ij} \right)^{2}}, \\ \end{aligned}\end{align*}

where $N_{s}$ is the number of spots, $N_{ct}$ is the number of cell types, $p$ is the cell-type proportion of ground truth and $q$ is the predicted proportion computed by $q_{ij}=\frac{N_{ij}}{N_{i}}$, where $N_{ij}$ denotes the number of cells of cell type $j$ on spot $i$, and $N_{i}$ denotes the number of cells on spot $i$, respectively. $L_{p}$ uses a RMSE metric not as a strict reconstruction loss, but as a soft constraint to guide the latent embeddings of $\boldsymbol{X}\boldsymbol{A}$ and $\boldsymbol{Y}$ toward a correlated structure. This allows the model to learn a mapping that preserves the relative expression trends across genes and spots, without forcing their absolute magnitudes to match, which would be invalid under batch effects.

The second term is the filtering regularity $L_{f}$, which constrains the output of DiSCO to have the structure of bipartite graph, i.e. the elements of the output are either 1 or 0: 


(12) \begin{align*}& L_{f} = \frac{1}{N_{c}\cdot N_{s}} \sum_{i=1}^{N_{c}} \sum_{j=1}^{N_{s}} \left(\boldsymbol{A}_{ij}-\boldsymbol{A}_{ij}^{2} \right).\end{align*}

The third term is the density regularity $L_{d}$, which constrains the learned density distribution of predicted spots is as similar as possible to the estimated density: 


(13) \begin{align*}& L_{d} = \operatorname{KL}\left(\frac{\boldsymbol{A}^{T}\mathbb{1}}{N_{c}}, \frac{\mathbb{1}}{N_{s}}\right),\end{align*}

where $\mathbb{1}$ represents all-ones vector, and $\operatorname{KL}$ denotes the **Kullback–Leibler divergence**, defined as: $\operatorname{KL}(P \| Q) = \sum _{x \in X} P(x) \,\ln \frac{P(x)}{Q(x)}.$

The term of $L_{d}$ encourages the model to learn a relatively uniform density and improves the generalization performance of the model.

In summary, the loss function is defined to train DiSCO as: 


(14) \begin{align*}& \begin{aligned} L &= L_{dpmm} + \lambda_{1} L_{p} + \lambda_{2} L_{f} + \lambda_{3} L_{d}, \\ \end{aligned}\end{align*}

where $\lambda _{1},\lambda _{2}$, and $\lambda _{3}$ are tuning parameters in the DiSCO model.

### Deconvolution

By employing discrete denoising steps conditioned on $\mathcal{D}=(\boldsymbol{X},\boldsymbol{Y})$, the trained DiSCO model directly infers the distribution of approximate solution $p(\boldsymbol{A})$ in the candidate solution space, and sample an approximate solution for the assignment. This process can be regarded as solving the Co problem $\arg \min _{\boldsymbol{A} \in \mathcal{A}} f(\boldsymbol{A}\mid \boldsymbol{X},\boldsymbol{Y} )$, which subsequently enables the cell type deconvolution task.

## Results

### Experimental setup

We use a 6-layer BGNN with a width of 256 as described above. All experiments were conducted on a system equipped with 4 NVIDIA GeForce RTX 4090 GPUs (24 GB), using CUDA 11.6, Python 3.9.18, and PyTorch 1.12.1. Training the DiSCO model took 47.5, with a peak system RAM usage of $\sim $14 GB. The final model comprises 1.7 million parameters, resulting in an estimated size of 6.982 MB.

In our training process, we intentionally add noise to the spatial transcriptomic data to enhance the model’s generalization performance, thereby mitigating the impact of batch effects between ST and SC data. The hyperparameters $\lambda _{1}$, $\lambda _{2}$, and $\lambda _{3}$ are all set to 1. Furthermore, to enhance the robustness of the model, we shuffled the gene inputs across tasks during the training process, while ensuring a one-to-one correspondence between the genes of the SC and the spot.

The test phase applies the pretrained DiSCO to deconvolute cell types for different simulated and real spatial transcriptome datasets. For ST across different datasets and the corresponding reference gene profiles, with $d$ denoting the number of shared genes, the following approach is taken: if $d$ is $\ge $2000, the top 2000 highly variable genes are selected; if $d$ is <2000, the BGNN node embedding layer utilizes the first $d$ model parameters. When fine-tuning is required, the loss used to update DiSCO is the cosine similarity between reconstructed and real gene profiles, the learning rate is set to $10^{-4}$, epoch is set to 30, the parameters from the final output layer of the network are employed for this process. The fine-tuned DiSCO is denoted as DiSCO*.

### Evaluation metrics

The quantitative metrics used for comparison with the ground truth include the RMSE and the Jensen–Shannon Divergence (JSD). Their calculation formulas are given below.

#### Root mean square errors

RMSE quantifies the difference in cell-type proportions between the predicted and ground truth values. Assuming that $N_{s}$ is the number of spots, $N_{ct}$ is the number of cell types, $p$ is the ground truth cell-type proportion, and $q$ is the predicted proportion, the RMSE can be expressed as: 


(15) \begin{align*}& \begin{aligned} L_{p} = \sqrt{\frac{1}{N_{s}\cdot N_{ct}} \sum_{i=1}^{N_{s}} \sum_{j=1}^{N_{ct}} \left(p_{ij}-q_{ij} \right)^{2}}. \\ \end{aligned}\end{align*}

#### Jensen–Shannon divergence

The JSD, also referred to as the JS distance, is used to measure the distance between two probability distributions. It can be computed as: 


(16) \begin{align*}& \operatorname{JS}(P \| Q) = \frac{1}{2} \operatorname{KL}(P \| M) + \frac{1}{2} \operatorname{KL}(Q \| M),\end{align*}

where $M$ is the mean of $P$ and $Q$: 


\begin{equation*}M = \frac{P + Q}{2}.\end{equation*}

### Simulation study

We tested 100 pairs of matched datasets from the simulation datasets by the pretrained DiSCO, and the results were compared with those of existing methods, including SD$^{2}$, DSTG, RCTD, Redeconve, Tangram, Cell2location.

**Figure 2:**
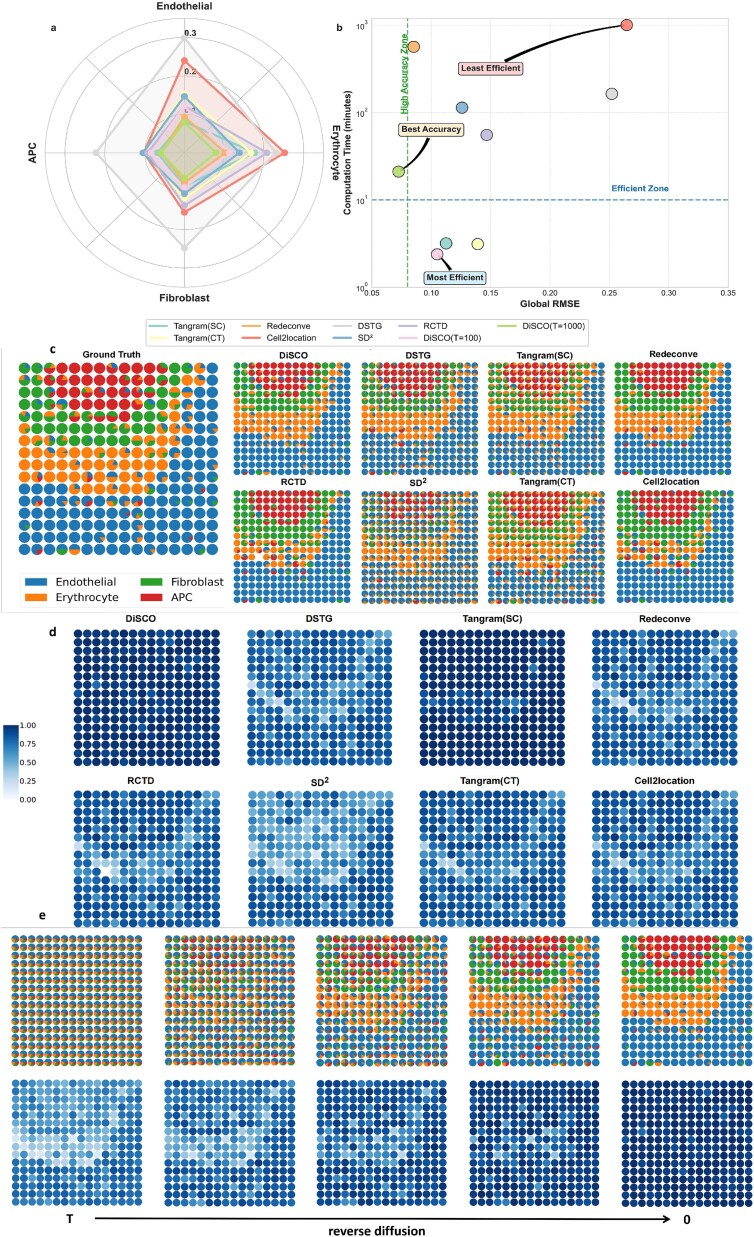
Visualization results for the simulation dataset. (a) The RMSE of 4 cell types on 100 paired simulation datasets. (b) The relationship between the total computation time and global rmse. (c) The spatial and cell-type distribution of the simulation dataset by different methods. (d) The cosine similarity between reconstructed gene profiles and ground truth for each spot. (e) The process of denoising on simulation data.

Figure 2a and Supplementary Table S1 reports the RMSE and JSD between the deconvolution results of various methods and the ground truth. For Tangram, SC denotes that the method uses a cell-to-spot mapping, CT denotes a cell type-to-spot mapping. For DiSCO, $T$ denotes the number of times our model has been inferenced about after acceleration in the reverse diffusion process. It is evident that at $T =100$, our results demonstrate superior performance compared to most of the methods. Furthermore, at $T =1000$, our results exhibit a significant lead over the other methods.

Figure 2b reports the computation time and global RMSE taken by each algorithm to complete $100$ instances of the deconvolution task. The results of DSTG and SD$^{2}$ include two parts: the first part is the time spent on generating pseudo-points, and the second part is the time spent both training on pseudo-points and testing on the actual spots. Figure 2b shows that our pretrained DiSCO model demonstrates superior speed while maintaining comparable accuracy to other methods. Notably, our model does not require updating gradients based on this test set, whereas other methods need to update their parameters based on the data of the current instance. Supplementary Fig. S1 further illustrates the trade-off between RMSE of the DiSCO deconvolution solution and the corresponding time cost across different inference durations. As inference time increases, RMSE decreases approximately linearly, while computational time rises progressively. This visualization aims to provide researchers with insights for balancing accuracy and time efficiency. In practice, users can refer to the curve in Supplementary Fig. S1 to select the inference step that aligns with their own accuracy/time constraints. This flexibility allows DiSCO to be adapted to different research scenarios without retraining the model.

Figure 2 further presents a visual example from the test set, where Fig. 2c visualizes the deconvolution results for each spot in that instance, and Fig. 2d displays the cosine similarity between the spatial transcriptome gene profiles obtained by the deconvolution results and the ground truth in each spot. As shown in Fig. 2c, our results effectively recover the cellular components of each spot when solving the example task with simulated data without any fine-tuning. This demonstrates that our pretrained foundational diffusion model can solve a series of CO problems in a feedforward manner without requiring task-specific gradient-based optimization at inference time, achieving strong generalization performance. In general, the pretrained DiSCO model yields more precise deconvolution results compared to other approaches, accurately predicting cell types in the majority of examined spots. Additionally, the proportion of cellular composition closely aligns with the actual values. In situations where the DiSCO model fails to accurately determine the cellular composition of certain spots, alternative methods also yield unsatisfactory solutions. Figure 2d illustrates that the spatial transcriptome gene profiles restored by our method exhibit a high degree of cosine similarity with the ground truth for each spot. This finding suggests that the discrepancy between the CO solution obtained by our algorithm and the optimal solution is negligible. Tangram(sc) achieves consistently high cosine similarities overall, while other comparative methods show relatively low cosine similarity in reconstructing the gene expression profiles of spots associated with the erythrocytes cell type. In contrast, our method demonstrates a markedly higher level of similarity in this region, outperforming all other approaches.

Figure 2e visualizes the reverse diffusion process for each spot. The upper panel illustrates the temporal evolution of cellular composition proportions within individual spots, while the lower panel quantifies the similarity between gene expression profiles computed from current cellular constituents and the ground truth. The deconvolution ground truth is provided in Fig. 2c. This demonstrates that cellular compositions progressively converge toward the ground truth during reverse diffusion, thus mechanistically explaining the transition from initial distributions to biologically relevant configurations at the spot level.

As a conditional generative model, DiSCO naturally supports uncertainty estimation for its deconvolution outputs. For each spot, the model provides not only the estimated cell-type proportions, but also confidence intervals for each proportion. Our estimation procedure follows three main steps: First, we perform multiple repeated samplings for each spot to obtain a series of deconvolution results; Subsequently, we treat these results as observations to compute moment estimates of the cell-type proportions per spot; Finally, we apply the bootstrap [21] method to construct confidence intervals for each proportion.

To validate this approach, we conducted an evaluation on a simulated dataset by performing 100 repeated deconvolution results for one representative task. The dataset comprises 252 spots and 4 cell types, yielding 1008 confidence intervals in total. The average width of the $95\%$ confidence intervals was 0.0141, reflecting high estimation precision. Comparison with ground truth revealed that 653 intervals ($64.78\%$) contained the corresponding true values. Most uncovered cases occurred when the true proportion was exactly $1$, due to the upper bound of the interval being slightly below 1 (e.g. [$0.999979, 0.999981$]). We further quantified the deviation for uncovered cases by computing the average distance between the ground truth and the nearest interval boundary, which was $0.0152$, confirming that the intervals remain tightly aligned with the ground truth.

### Real spatial transcriptomics datasets

We evaluate the pretrained DiSCO on two SC resolution datasets, Mouse Brain Cortex on seqFISH+ [3], Mouse Hypothalamic Preoptic on MERFISH [4], and two spot resolution datasets, PDAC data [22], Human Breast Cancer data on Visium $10\times $. For the first two SC resolution datasets, different spots were artificially divided based on SC locations to reduce the resolution, similar to the procedure used for generating the simulation dataset to obtain low-resolution data. Concurrently, matched reference SC datasets were obtained along with low-resolution ST data. For the last two spot resolution datasets, due to the lack of ground truth for cell types in the spots, we only performed qualitative analysis on both datasets.

#### Evaluation on mouse brain cortex

We collected ST data and reference data of the mouse brain cortex, with ST data achieving SC resolution using seqFISH+ [3]. The dataset includes 523 cells, encompassing 6 cell types: excitatory neurons (eNeurons), inhibitory neurons (iNeurons), astrocytes, oligodendrocytes (Olig), microglia, and endothelial-mural cells (endo-mural). The cells are divided into spots of varying resolutions ($0.5\times , 1\times $, and $2\times $) based on patches with different window sizes ($25.75\,\mathrm{\mu} $m, $51.5\,\mathrm{\mu} $m, and $103\,\mathrm{\mu} $m). We evaluated DiSCO on the Mouse Brain Cortex data at ($0.5\times , 1\times $, and $2\times $) resolutions, respectively.

**Figure 3:**
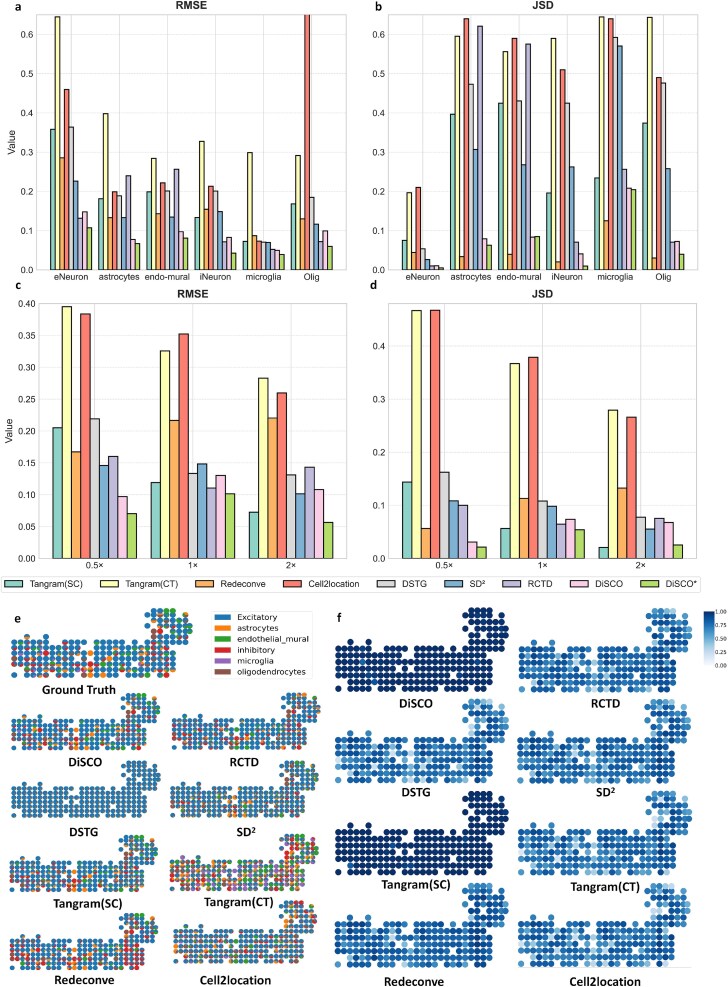
Visualization results of deconvolution on Mouse Brain Cortext. (a) The RMSE of 6 cell types on Mouse Brain Cortex ($0.5\times $). (b) The JSD of 6 cell types on Mouse Brain Cortex($0.5\times $). (c) The global rmse on Mouse Brain Cortex. (d) The global JSD on Mouse Brain Cortex. (e) The spatial and cell-type distribution of each spot with different methods. (e) The cosine similarity between reconstructed gene profiles and ground truth for each spot.

Figure 3a and b and Supplementary Figs S2 and S3 illustrate the performance of the deconvolution results of each algorithm on different cell types, respectively. Figure 3c and d demonstrates the global performance of the algorithms on this task instance, where * indicates that the algorithms are fine-tuned on this dataset. It is evident that our algorithm can achieve above-median performance across all metrics without the need for fine-tuning. However, DiSCO was applied in a zero-shot manner without any prior exposure to brain data, the above-median performance under this challenging condition validates DiSCO’s strength as a foundational model. Furthermore, as the resolution increases, our method shows significant improvement in the recognition of cell types such as astrocytes, iNeurons, and microglia. These findings demonstrate that our method is more advantageous when applied to higher resolution data without the necessity for fine-tuning. Moreover, upon fine-tuning the model, a more pronounced advantage is observed in both overall and specific cell type classifications across both high-resolution and low-resolution datasets.

As illustrated in Fig. 3e and f, the deconvolution results and cosine similarity of each method for each spot on Mouse Brain Cortex at ($0.5\times $) data are demonstrated. In Fig. 3e, DiSCO* successfully deconvolutes a satisfactory number of cell types. Although there are some incorrectly predicted cell types in a few spots, they constitute a very small proportion of the respective spots. Furthermore, the proportion of cell types in each spot closely aligns with the ground truth. In contrast, alternative methods frequently result in the erroneous prediction of cell types and overestimate incorrectly predicted cell types in the respective spots. It matches the result of Fig. 3f, as DiSCO reconstructs spot profiles with high similarity to the ground truth. The deconvoluted results of DSTG reveals a high degree of homogeneity among the spots, with each spot exhibiting insignificant variation. This phenomenon can be attributed to the Mouse Brain Cortex dataset containing a significant proportion of excitatory cell types and having an inadequate slice size, resulting in a limited number of spots and overfitting during the training process. The SD$^{2}$ method demonstrates a modest improvement over method DSTG, it also exhibits signs of homogenization. This finding suggests that methods for generating pseudo-spots are more dependent on larger, relatively balanced datasets in terms of cell types.

**Figure 4:**
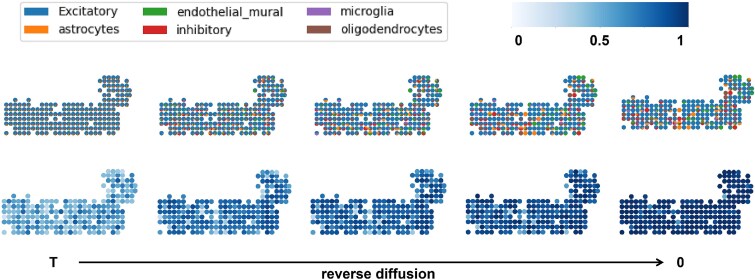
Visualization results of deconvolution on Mouse Brain Cortext for reverse diffusion process. The upper half of the figure represents the cell type proportions, while the lower half shows the similarity between the generated ST profile and the ground truth.

**Figure 5:**
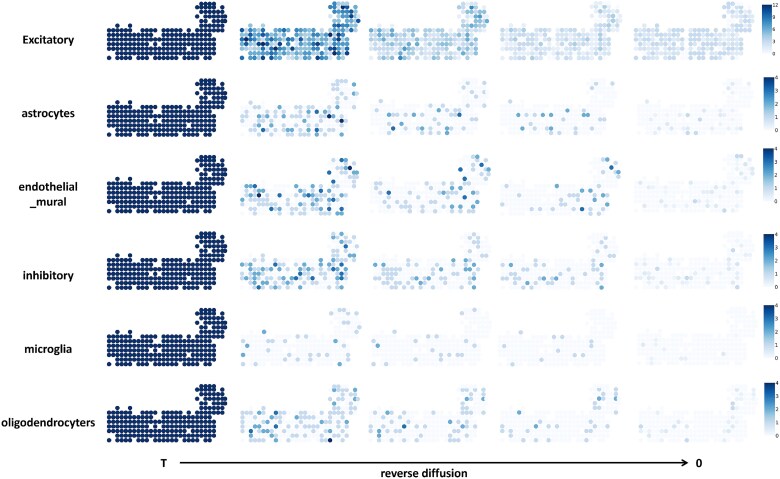
Visualization results of cellular abundance on Mouse Brain Cortext for reverse diffusion process. Each row shows the specific cellular abundance. From left to right, it illustrates the optimization process of DiSCO in solving the deconvolution problem.

Figures 4 and 5 visualize the reverse diffusion process of DiSCO applied to the Mouse Brain Cortex for ST deconvolution. Figure 4 depicts the evolving cell type proportions and the similarity between the generated spatial transcriptomic profile and the ground truth throughout this process. In the early reverse diffusion stage, each spot is dominated by excitatory neurons, and the overall cell type distribution remains relatively uniform across spots, both driven by the composition inherited from the referenced SC profiling. As reverse diffusion proceeds, the proportions of each cell type within spots gradually transform to more biologically plausible levels and begin to exhibit enhanced spatial coherence. Concurrently, the similarity between the reconstructed spatial transcriptomic profiles and the ground truth improves steadily, confirming that the process recovers meaningful structure. Figure 5 illustrates the dynamics of cell abundance for each cell type during reverse diffusion. Initially, each spot contains an overestimated number of cells across all types, resulting from the initial assignment of SCs from the reference dataset to each spot at a fixed $0.5$ ratio. At this stage, the combined cell abundance and type proportions match the distribution sampled by DiSCO at time $T$. As the reverse diffusion advances, the estimated count of each cell type per spot decreases, and distinct spatial heterogeneity gradually emerges. This emerging heterogeneity shows a strong correlation with the heterogeneity observed at time $0$. These intermediate states are not merely algorithmic artifacts; they offer a principled, stepwise view of how cellular composition and spatial organization are jointly inferred. By tracing this trajectory, researchers can gain heuristic insight into potential biological processes, such as local cell dispersion, density regulation, or niche formation, which underlie the observed spatial patterns.

#### Evaluation on mouse hypothalamic preoptic

We collected ST data and the referenced data of mouse hypothalamic preoptic by MERFISH [4]. MERFISH is an imaging-based cell type identification and mapping method that can achieve SC resolution. The dataset under consideration discloses the hypothalamic preoptic region of different mice. It comprises 36 samples totaling 181 slices, from which we selected all slices from the 26th mouse for the experiment. Each slice contains 160 genes and 8 cell types, with 5150 and 5141 cells, respectively. Current sequencing technologies also generate substantial volumes of such small number gene data, presenting significant challenges for deconvolution. The robustness of our model can be fully verified using such a dataset with a small number of genes.

Similar to the simulation procedure in the Mouse Brain Cortex dataset, we binned the squares of $75\times 75$ cells as one spot in the MERFISH datasets, producing 556, 575 spots with their real cell-type proportions, respectively. Since 2000 genes were used in the DiSCO training process, we selected the parameters of the first 160 sets of models when embedding the SCs as well as the spots.

**Figure 6:**
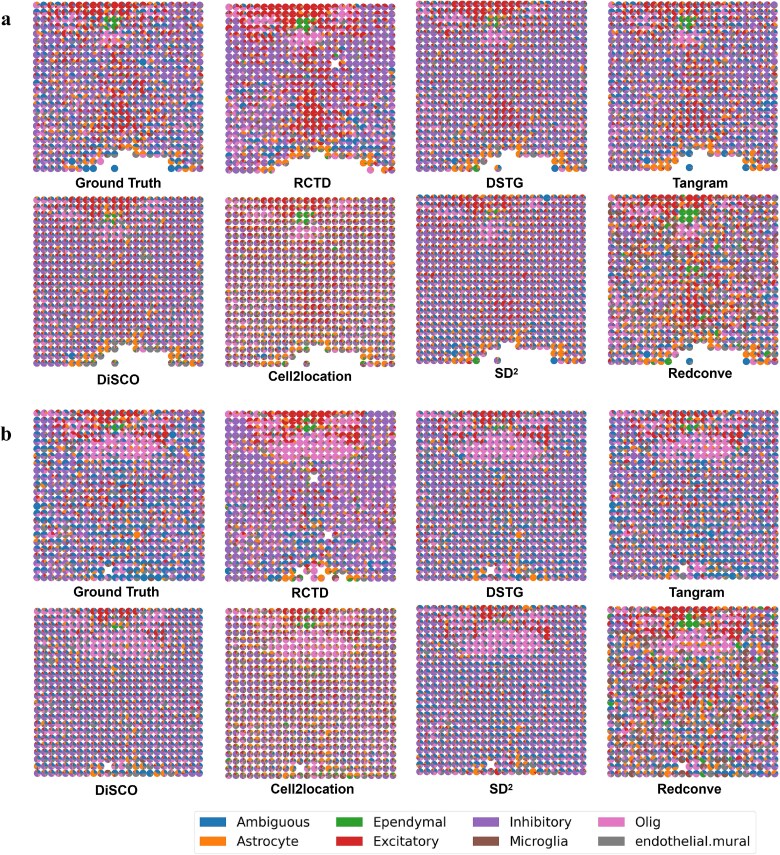
Deconvolution results for the first (a) and second (b) slices of the 26th mouse in the Mouse Hypothalamic Preoptic data.

We visualized the deconvolution results of DiSCO and compared methods across all slices in Fig. 6, Supplementary Figs S4 and S5. As shown, the cellular composition predicted by DiSCO for each spot closely matches the ground truth and reveals a strong spatial concordance across tissue sections. DiSCO achieved competing visual and quantitative performance compared with other methods, demonstrating that DiSCO exhibits robust generalization performance, even when processing data with a limited number of genes.

#### Evaluation on PDAC data

We also evaluated DiSCO on the PDAC (pancreatic ductal adenocarcinoma) dataset [22]. PDAC is a highly devastating and heterogeneous disease in humans, characterized by poor prognosis and rising incidence. The ST data of PDAC were generated using the original ST method, with a low resolution of $\sim $10–40 cells per spot. The PDAC-A dataset includes 428 spots with 20 cell types, and the paired SC RNA sequencing data were generated from pancreatic adenocarcinoma tissue using the InDrop technique [23], consisting of 1926 cells.

**Figure 7:**
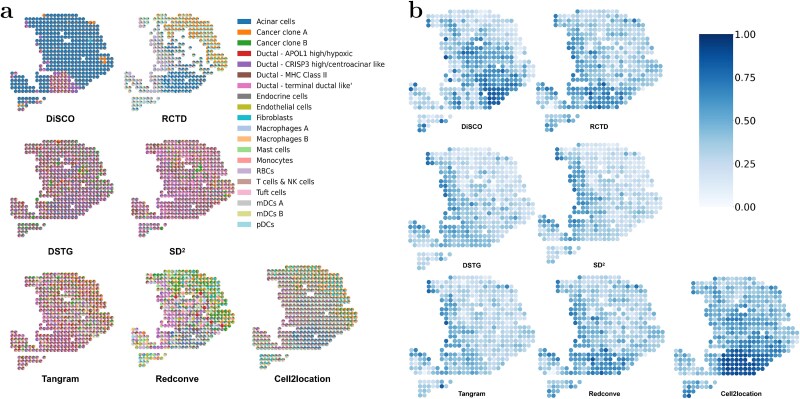
Visualization results on PDAC-A. (a) The cellular components of each spot with different methods. (b) The cosine similarity between reconstructed gene profiles and ground truth for each spot.

Figure 7 presents the results of DiSCO on the PDAC-A dataset. Figure 7b shows that cosine similarity is generally limited across all methods. Nevertheless, DiSCO achieves better performance than many alternatives in these spots, and provides results complementary to RCTD, enhancing the overall biological interpretability. Conversely, although RCTD achieves high deconvolution similarity at certain spots, it severely suffers from low sensitivity, failing to detect cells in a substantial fraction of spots. Furthermore, it inherently requires a large number of cells per type, a condition often unmet in real datasets. As a result, its performance is frequently limited in practice. DiSCO was developed to overcome these constraints and perform robustly even in scenarios with low cell counts.

#### Evaluation on human breast cancer data

**Figure 8:**
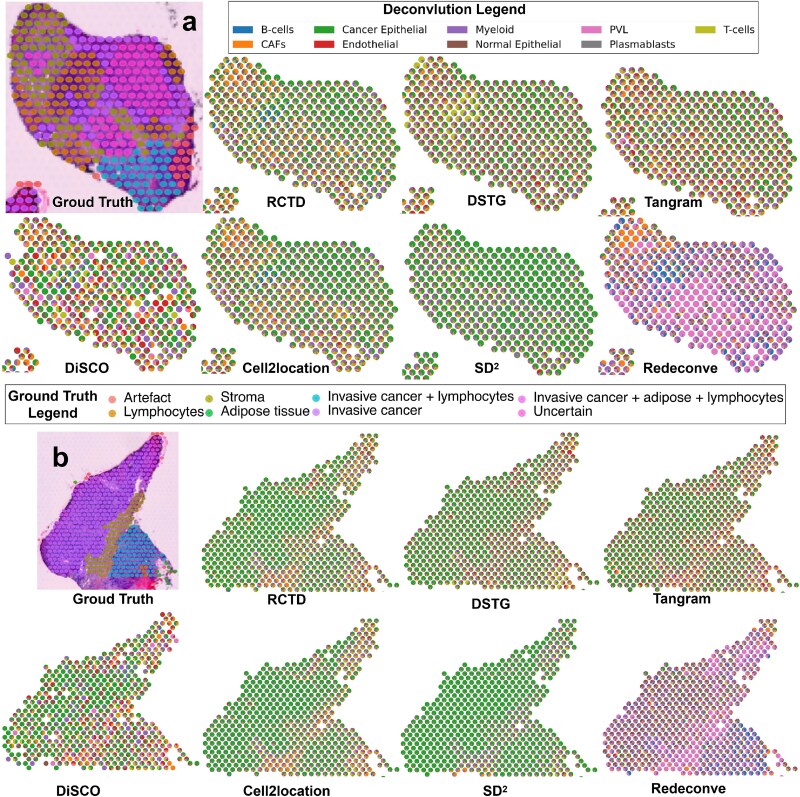
Visualization results on Human Breast Cancer data. (a) The cellular components of each spot on “CID4535 a.” (b) The cellular components of each spot on “CID4535 b.”

We collected ST data and reference data of the Human Breast Cancer on Visium $10\times $. Sample (“CID4535,” ER+) contains 1127 tissue spots across three regions (a, b, c). We used matched reference scRNA-seq data (GSE176078; 100 064 cells). Region c was excluded from quantitative analysis due to its uniform pathological annotation. We therefore focused on regions a and b. As shown in Fig. 8, DiSCO performs better than other methods when using only 2000 genes. In region a, DiSCO clearly identifies Endothelial cells within and around the “Invasive cancer + lymphocytes” area, alongside Perivascular-like (PVL) cells. Cancer Epithelial cells are correctly located in tumor regions, where T cells are also appropriately co-localized. In region b, DiSCO appropriately shows very few Cancer Epithelial cells on the Stroma area. This matches the expected histology. Other cell types in this region also show clear spatial patterns. In contrast, other methods gave results where almost every spot contained all cell types. These results show little spatial structure and are less biologically meaningful.

### Ablation study of DiSCO

**Figure 9:**
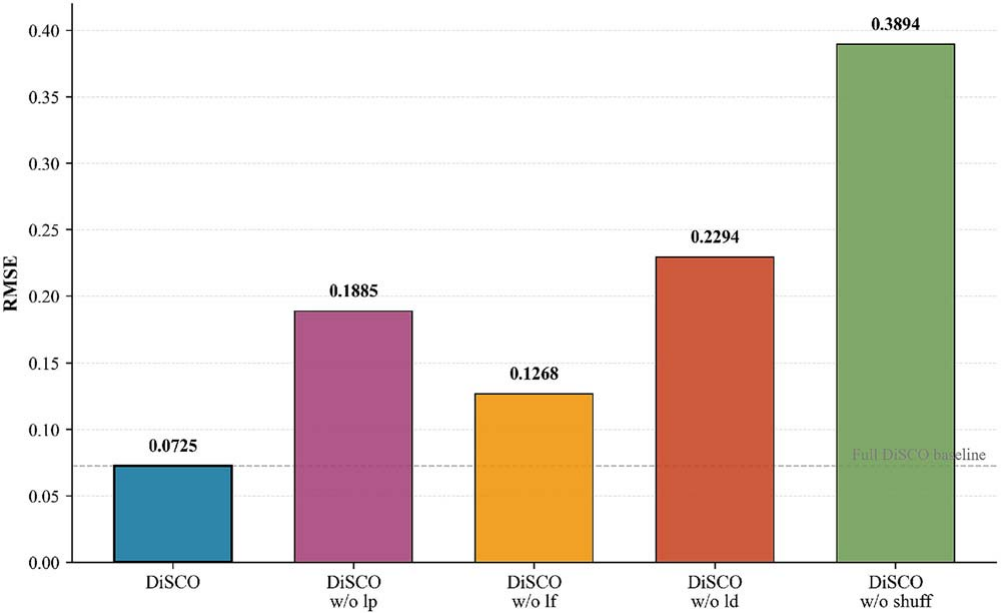
Visualization for ablation study, where “w/o” indicates training the model while omitting the specified component.

Figure 9 illustrates the ablation experiments on simulation data by DiSCO algorithm. The notation “w/o” indicates training the model while omitting the specified component: “lp” “lf,” and “ld” correspond to the three regularization loss terms introduced in Equation (14), whereas “shuffle” refers to randomly permuting the order of genes during training. The ablation study clearly demonstrates the contribution of each regularization term. As shown in the Fig. 9, removing any of the three loss components leads to a noticeable increase in RMSE: specifically, the term $L_{d}$ has the largest impact, confirming its importance in recovering realistic cellular distributions. The term $L_{f}$ and term $L_{p}$ also provide significant and complementary improvements in deconvolution accuracy. Furthermore, we tested a “shuffle” baseline where gene order is randomized during training. Its poor performance confirms that meaningful gene shuffle is crucial, and that our model successfully learns structured patterns rather than overfitting to arbitrary inputs.

## Discussion

In this work, we proposed a foundational deconvolution model named DiSCO. The deconvolution task is formulated as a CO problem with 0-1 assignments. The DiSCO enables to learn a generalizable optimization solver for a series of task-specific CO problems derived from cell type deconvolution tasks.

The pretrained DiSCO model can be applied directly to various datasets for cellular deconvolution tasks. Comprehensive experimental evaluation was conducted to benchmark the performance of DiSCO against other deconvolution methods. The empirical results indicate that DiSCO consistently achieves competing performance across a range of quantitative metrics without requiring fine-tuning. Upon fine-tuning, DiSCO could obtain superior performance on datasets with varying resolutions and gene numbers.

Despite DiSCO’s commendable performance, there are several areas for improvement. First, DiSCO currently focuses solely on individual gene profiles during the deconvolution process, without incorporating spatial relationships between spots or the topological relationships among cells. Second, the training data used for DiSCO are exclusively generated from scCube, which may limit its generalizability to some extent. Third, DiSCO’s generalization can be challenged when the available gene set for cross modality alignment is very limited, reducing the signal for accurate mapping. Future work will focus on optimizing DiSCO by addressing these limitations.

**Key Points**

- We formulate the deconvolution of ST data as a task-specific deconvolutional combinatorial optimization (CO) problem, and design the DiSCO as the CO solver to address the cell type deconvolution.
- We introduce a foundational diffusion model, which can help learn a generlizable optimization solver for a series of task-specific CO problems derived from cell type deconvolution tasks. Subsequently, the trained DiSCO model can be applied directly to various datasets for novel cellular deconvolution tasks.
- We empirically validated DiSCO by using both simulated datasets and real datasets, demonstrating that the pretrained DiSCO model performs effectively and efficently on datasets with varying resolutions and different numbers of genes, thus highlighting its capacity to effectively generalize to diverse datasets.

## Supplementary Material

Supplementary_bbag207

## Data Availability

Mouse Brain Cortex is collected from Gene Expression Omnibus with accession number GSE98674. Mouse Hypothalamic Preoptic is collected from MERFISH. PDAC and the matched reference are all available on GSE111672. The file of the ST data we used was “GSM3036911_PDAC-A-ST1-filtered.txt.gz,” and the file of the scRNA-seq data we used was “GSE111672_PDAC-A-indrop-filtered-expMat.txt.gz.” Human Breast Cancer was available 10x with sample “CID4535.” The referenced scRNA-seq data that includes 100 064 SCs with annotations are available on GSE176078.
