# Supplementary material for: DiSCO: deconvoluting spatial transcriptomics via combinatorial optimization with a foundational diffusion model
Source: Brief Bioinform. 2026 May 8;27(3):bbag207. doi: 10.1093/bib/bbag207 (PMC13155122; doi:10.1093/bib/bbag207)
Supplement: Supplementary_bbag207 [file supplementary_bbag207.pdf]

Supplementary Materials

Table 1: JSD of different methods across cell types on simulation data.

	**Endothelial**	**Erythrocyte**	**Fibroblast**	**APC**	**global**
**Methods**					
Tangram(SC)	0.011400	0.017690	0.016800	0.028000	0.021250
Tangram(CT)	0.154300	0.159000	0.168400	0.106200	0.189100
Redeconve	0.139324	0.231476	0.154010	0.099575	0.163638
Cell2location	0.024000	0.045210	0.035200	0.050180	0.066400
DSTG	0.029900	0.093070	0.024800	0.022230	0.035200
SD^2	0.014700	0.138500	0.020660	0.030420	0.025900
RCTD	0.012210	0.040750	0.013750	0.092600	0.019670
DiSCO	0.012150	0.041240	0.039800	0.033700	0.024000
DiSCO*	0.008889	0.024838	0.010452	0.017362	0.015669

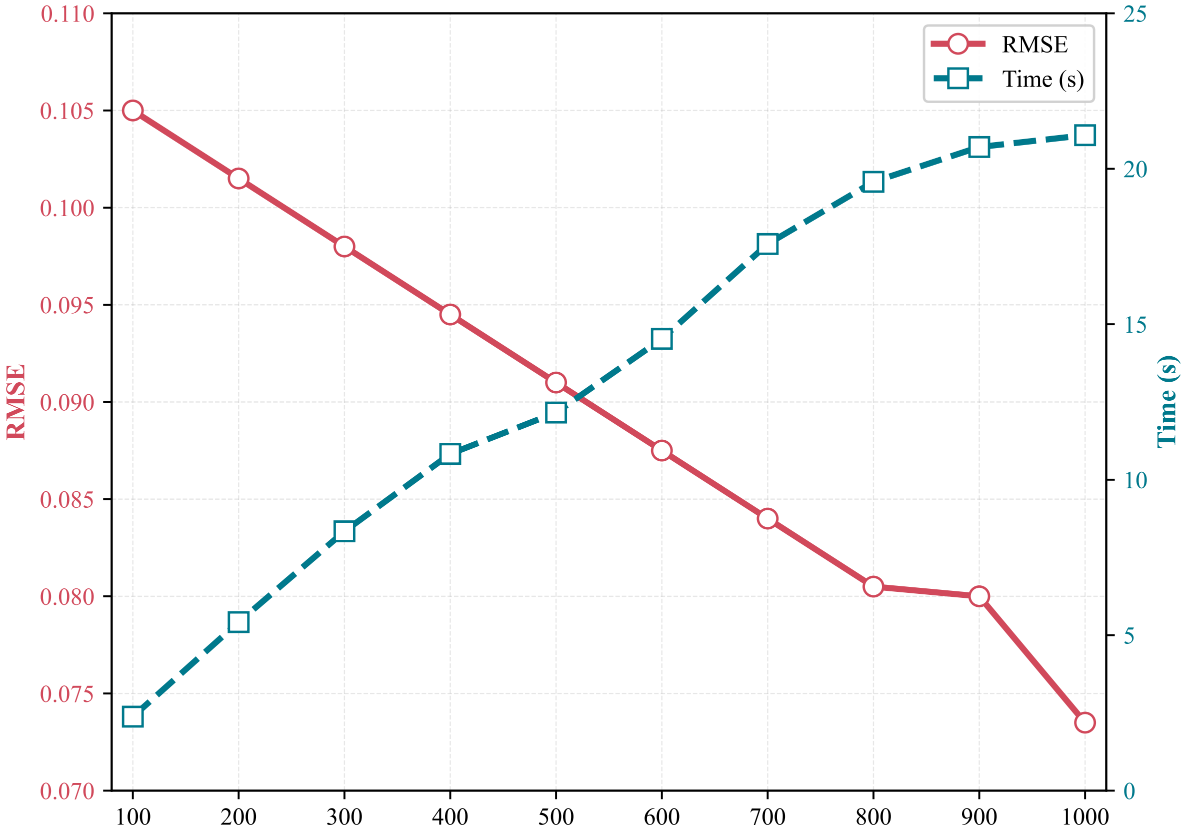

Figure 1: The performance for DiSCO on different inference step.

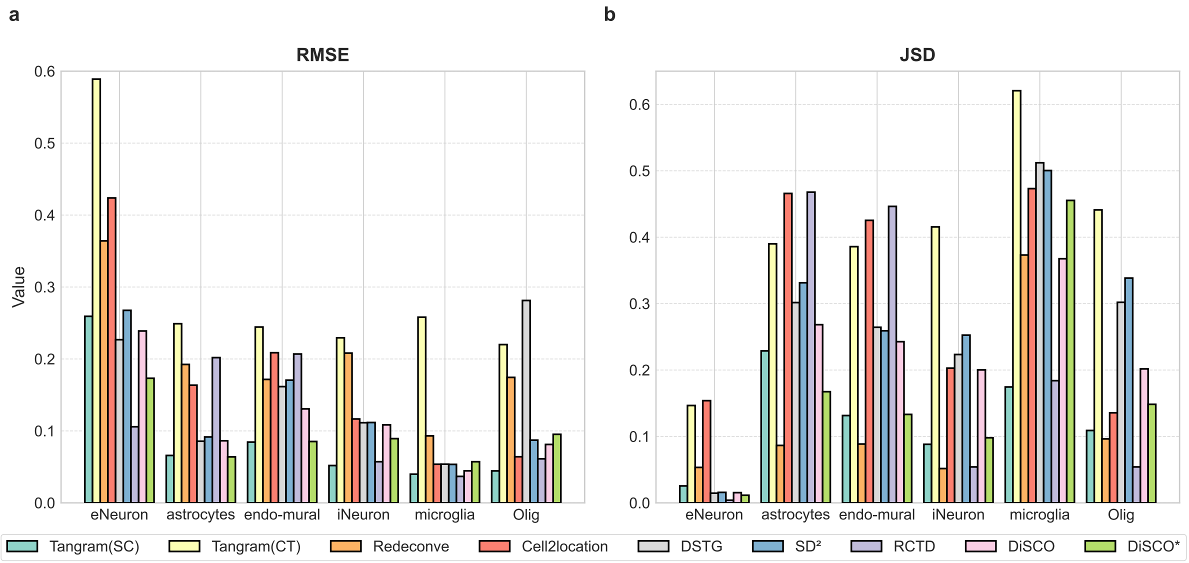

Figure 2: Visualization results on Mouse Brain Cortex(1×).**a** The first figure shows the spatial and cell-type distribution, and the rest are the cellular components of each spot with different methods. **b** The cosine similarity between reconstructed gene profiles and ground truth for each spot.

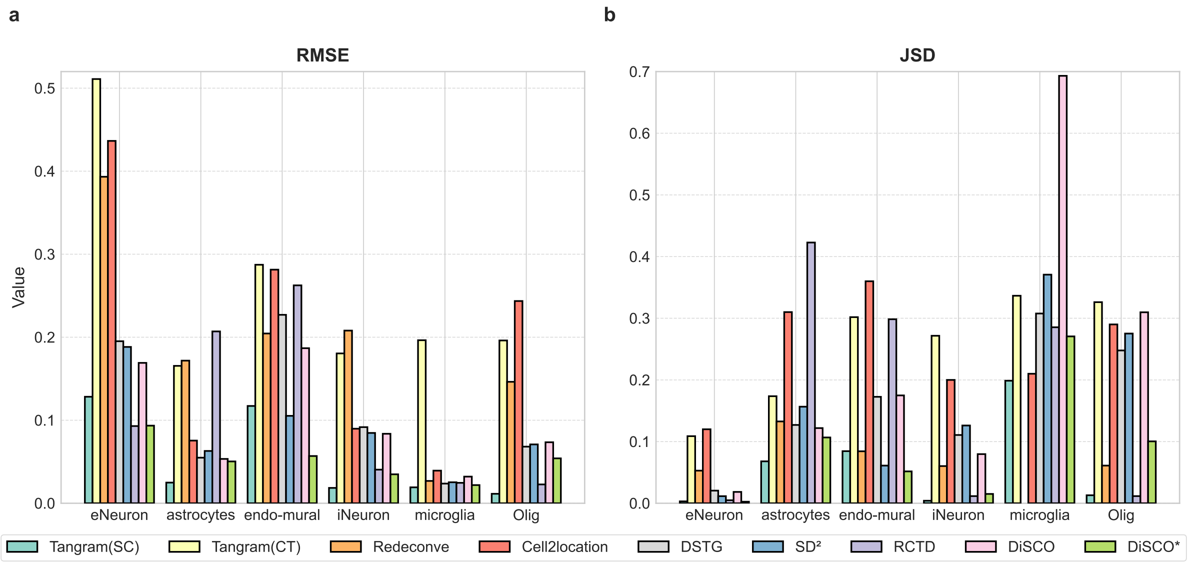

Figure 3: Visualization results on Mouse Brain Cortex(2×).**a** The first figure shows the spatial and cell-type distribution, and the rest are the cellular components of each spot with different methods. **b** The cosine similarity between reconstructed gene profiles and ground truth for each spot.

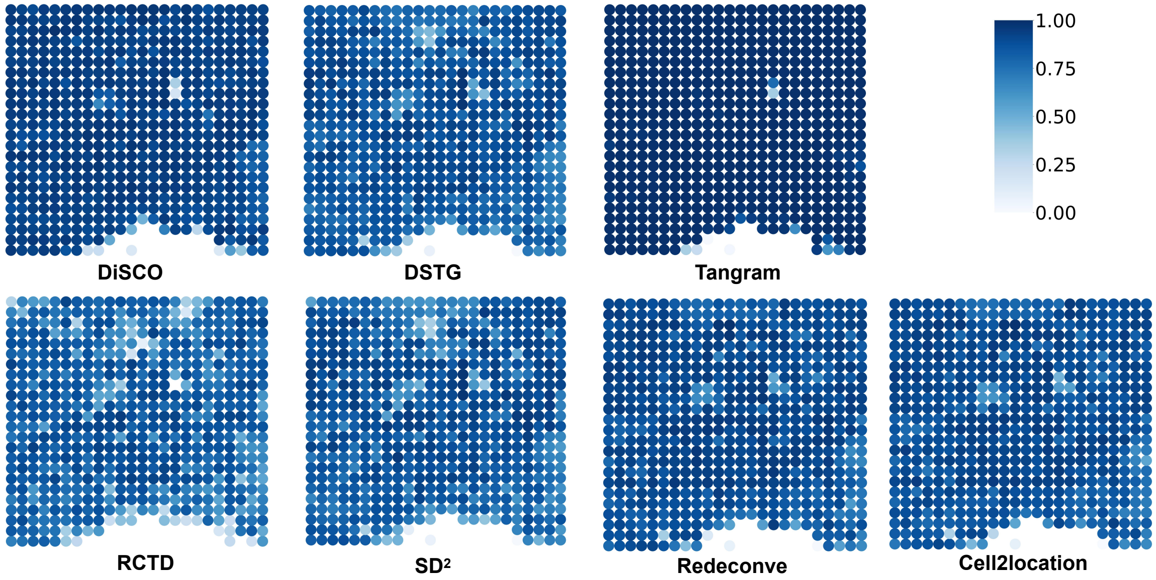

Figure 4: Cosine similarity results for the slice of the 26th mouse in the Mouse Hypothalamic Preoptic data.

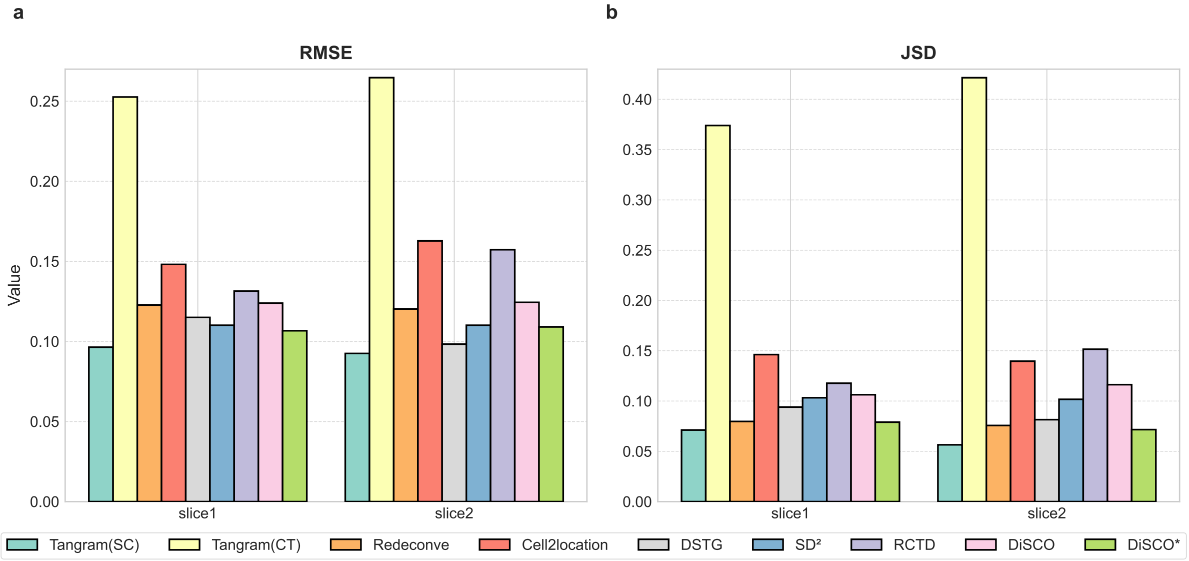

Figure 5: RMSE results for the slice of the 26th mouse in the Mouse Hypothalamic Preoptic data.
